# Detection of Potato spindle tuber viroid sequence variants derived from PSTVd-infected *Phelipanche ramosa* in flower organs of tomato plants

**DOI:** 10.1080/13102818.2014.918709

**Published:** 2014-07-04

**Authors:** Tihomir Vachev, Desislava Ivanova, Galina Yahubyan, Samir Naimov, Ivan Minkov, Mariyana Gozmanova

**Affiliations:** ^a^ Department of Plant Physiology and Molecular Biology, University of Plovdiv, Plovdiv, Bulgaria

**Keywords:** PSTVd, PSTVd sequence variants, *P. ramosa*, flower organs

## Abstract

Potato spindle tuber viroid (PSTVd) is an infectious small, circular, non-coding single-stranded RNA that induces disease on many crop species, ornamental plants, weeds and parasitic plants. PSTVd propagate in their host as a population of closely related but non-identical RNA variants referred to as quasispecies. Recently, we have described three *de novo* arising PSTVd variants in the parasitic plant *Phelipanche ramosa* after mechanical inoculation with the PSTVd KF440-2 isolate. These *P. ramosa* derived mutants were designated as G241-C, C208-U and C227-U PSTVd variants. Each of these variants carries a single-nucleotide substitution compared to the PSTVd KF440-2 sequence from which they are considered to have evolved. Here we complement our previous studies on these mutants by exploring their potential to infect the floral organs of tomato plants. We found that the PSTVd G241-C and C208-U variants were able to replicate in systemic leaves and floral organs of tomato plants, while the PSTVd C227-U variant did not develop systemic infection. Furthermore, we analysed the progeny of these PSTVd variants in sepals and petals of tomato plants for retention of the specific mutations.

## Introduction

Potato spindle tuber viroid (PSTVd) is a representative member of family *Pospiviroidae*.[1] Its genome consists of a 359-nt-long non-coding single-stranded RNA, which adopts a rod-like conformation with five distinct domains (from the 5′ terminal end: left (TL), pathogenic (P), central conserved region (CCR), variable (V) and terminal right (TR) domain) built of (paired) stems and (unpaired) loops.[2] The significance and role of more than one domain in a particular function have been reported. However, certain PSTVd structural elements (virulent modulating (VM) region in the P domain; RY motif in the TR domain) or temporally acquired metastable structures (HPII) have been associated with specific functions.[3]

PSTVd replicates in the nucleus via the asymmetric rolling circle pathway of multiplication; it exploits the host RNA polymerase II, host cleavage factors and host ligase for its life cycle.[4,5] PSTVd replicates and moves to sink, but not source, leaves.[6] It was absent from shoot apical meristems. In the flowers of mechanically infected tomato and *Nicotiana benthamiana* plants, PSTVd was present in the sepals, but was absent in the petals, stamens and ovary.[6] This observation was attributed to restricted traffic of PSTVd into these organs and not to suppression of replication.[7] However, our preliminary analysis showed that the PSTVd KF440-2 isolate could infect both sepals and petals of mechanically infected tomato plants.[8] Successful replication of PSTVd in all floral organs was reported also in transgenic *N. benthamiana* that expressed cDNA of PSTVd intermediate strain under the 35S promoter.[7]

PSTVd propagates as a population of closely related but not identical RNA variants. The variants appear due to the high rate of replication, disabled error prone activity of RNA polymerase II and selective pressure of the host.[9–13] The effect of sequence variations on disease expression was studied in a pool of single-point mutants,[14–17] ‘thermomutants’ [9] or in libraries of mutants with genomes partially or fully randomized at certain positions (*in vivo* Selex) [12]. It was found that the mutations affected either replication or movement of the PSTVd.[10–12,18] Thus, many mutations were identified as stably maintained and preserved in the progeny,[17] while others were identified as untolerated in a given host and were substituted by changes in different positions of the genome or by reversion to the wild-type sequence.[10,13,19–21]

Characterization of new PSTVd variants emerging as a result of adaptation to alternative hosts might help for further determination of specific nucleotide changes that contribute to the systemic infectivity of PSTVd.[22] Recently, we reported that the parasitic plant *Phelipanche ramosa* was able to sustain replication of PSTVd.[23] The analysis of progeny derived from mechanical inoculation of the stem of *P. ramosa* with the KF440-2 isolate revealed the presence of three variants, with mutations located in the lower strand of the C domain (PSTVd G241-C), in the lower part of the RY motif of the TR domain (PSTVd C208-U) and in the lower part of the V domain (PSTVd C227-U).[23] The secondary structure of these variants was analysed and the possible conformation changes were predicted by an *in silico* RNA prediction tool (MFold).[24] Bioassays on Rentita tomato plants showed that the PSTVd G241-C and PSTVd C208-U mutants replicated in tomato plants, while PSTVd C227-U did not infect the experimental host.[23] In this study, we complement the current knowledge about these *P. ramosa* PSTVd variants by exploring their capability to move from an inoculated tomato leaf towards tomato floral organs and to propagate in them.

## Materials and methods

### Plant material and inoculation of plants

Tomato plants (*Solanum lycopersicum*) cv. Rentita were grown in a green chamber at 28 °C, at a 14/10 h light/dark cycle. Tomato seedlings were mechanically inoculated with 250 ng of the PSTVd KF440-2 isolate or *P. ramosa* PSTVd (G208-U, C227-U and G241-C) variants transcribed *in vitro*. Mock-inoculated plants were treated with 1% potassium phosphate buffer.

### Plasmid constructs

*P. ramosa* PSTVd variants G208-U, C227-U and G241-C were cloned in a pCRII TOPO cloning vector (Invitrogen). BamHI tailed PSTVd oligos (Fw 5′-AGG GAT CCC CGG GGA AAC CTG GAG CGA-3′; Rev 5′-GGG GAT CCC TGA AGC GCT CCT CCG AGC-3′) were used for subcloning of the PSTVd variants in a pHa106 plasmid.

### *In vitro* transcription

The longer-than-unit-length PSTVd(+) RNA of the KF440-2 isolate and each *P. ramosa* variant (G208-U, C227-U and G241-C) were obtained by *in vitro* transcription of EcoRI-linearized pHa106 plasmids, using Sp6 RNA polymerase.[25] Approximately 1 μg of *Hind*III-linearized pHa106 plasmid was used as a template for *in vitro* transcription of longer-than-unit length PSTVd(−) RNA of the KF440-2 isolate synthesized with T7 RNA polymerase. Digoxigenin (DIG)-labelled PSTVd(−) RNA was prepared with DIG-labelled Uridine triphosphate (UTP), following the instructions of the DIG Northern Starter Kit (Roche).

### RNA extraction

Total RNAs were extracted from systemic tomato leaves at 28 days post inoculation (d.p.i) and from floral organs at 65 d.p.i as previously described.[26] The quality of RNA preparations was checked in a 1% agarose gels stained with ethidium bromide and visualized under ultraviolet (UV) light. The RNA quantity was determined spectrophotometrically.

### Northern blot analysis

Total RNA isolated from floral organs and systemic leaves (5 μg) was separated in a 1.4% formaldehyde agarose gel. The gel was transferred to a Hybond-XL nylon membrane (Amersham Biosciences) overnight by capillary transfer, using 2 × saline sodium citrate buffer (SSC), and the RNA preparations were immobilized by UV cross-linking. The membrane was stained with methylene blue and then prehybridized with sodium dodecyl sulphate (SDS) hybridization buffer (7% SDS, 50% deionized formamide, 5 × SSC, 2% blocking reagent, 0.1% (w/v) N-laurylsarcosine, 50 mmol/L sodium phosphate, pH 7.0) at 68 °C for 1–2 h. Hybridization was carried out overnight at 68 °C in the same buffer with the addition of DIG-labelled PSTVd(−) RNA probe. Then, the membrane was washed twice in 2 × SSC/0.1% SDS for 5 min at room temperature, followed by two more washes in 0.1 × SSC/0.1% SDS for 15 min at 68 °C. The detection was performed with an anti-DIG-antibody (Ab) conjugated to alkaline phosphatase and visualized with nitroblue tetrazolium (NBT)/5-bromo-4-chloro-3-indolyl phosphate ready-to-use tablets (Roche).

### Reverse transcription polymerase chain reaction (RT-PCR)

RT-PCR was used for cDNA synthesis of genomic and anti-genomic PSTVd strands. One microgram of total RNA obtained from sepals and petals of infected and mock-inoculated tomato plants was used for first-strand cDNA synthesis (USB corporation (USB)). To synthesize cDNA specific for PSTVd, the primers described by Weidemann and Buchta [27] were used: cDNA specific for the genomic PSTVd(+) strand was synthesized with PSTVd reverse primer (5′-CCCTGAAGCGCTCCTCCGAG-3′), while the cDNA specific for the antigenomic PSTVd(−) strand was obtained with the forward primer (5′-ATCCCCGGGGAAACCTGGAGCGA-3′). The template RNA was removed by additional ribonuclease (RNase H) treatment at 37 °C for 1 h.

### Cloning and sequencing of PSTVd variants from petals and sepals

For sequencing purposes, a PSTVd-specific PCR reaction was set up using Vent DNA polymerase (New England Biolab). Amplification products were cloned into a pCRII TOPO vector system (Invitrogen), according to the manufacturer's instructions. Two independent randomly selected clones derived from sepals and petals were analysed by bidirectional sequencing (T7 and SP6 promotor primers) (AGOWA, Germany).

### Trypan blue staining

Cross sections of tomato leaves infected with the PSTVd KF440-2 isolate and *P. ramosa* variants (G241-C, G208-U, C227-U) were treated with lactophenol–trypan blue solution (10 mL of phenol, 10 mL of 100% glycerol, 10 mL of lactic acid, 0.02 g of trypan blue, 10 mL of water mixed with 96% ethanol (1:2)) and boiled for 2 min. Then, the specimens were incubated for 1 h at room temperature in the same solution. The slices were destained in chloral hydrate solution (pH = 1.2). Total destaining was achieved in approximately 24 h. Observation of leaf necrosis was performed with light microscopy in 50% glycerol.

## Results and discussion

### Detection of leaf necrosis in tomato plants

**Figure 1.**
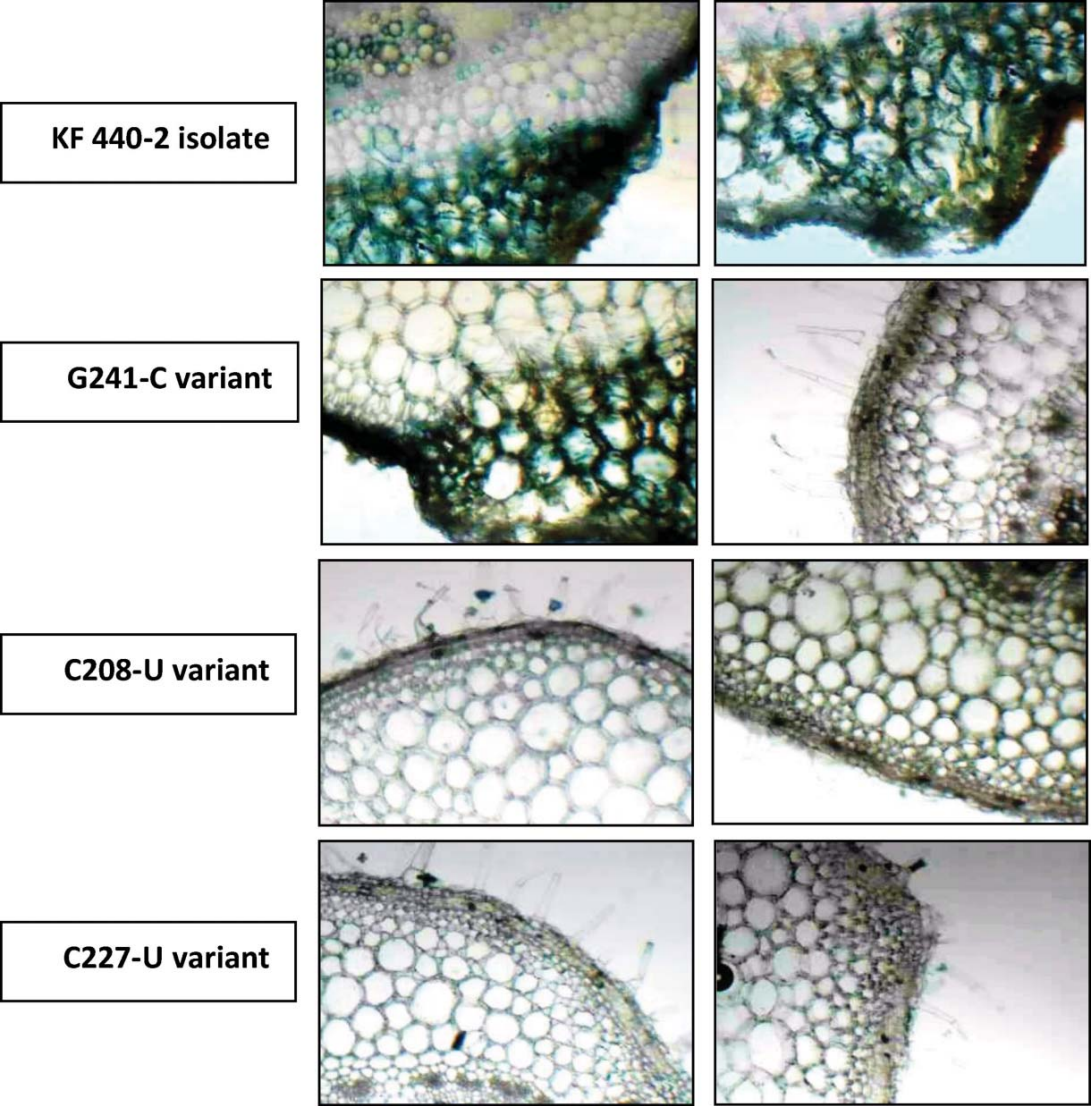
Leaf necrosis caused by PSTVd infection on tomato plants. Trypan blue staining of cross sections of leaf tissue from tomato plants infected with PSTVd KF440-2 and *P. ramosa* variants.

PSTVd symptoms could vary from mild to lethal depending on the host–PSTVd strain combinations and environment conditions. The main symptoms expressed by sensitive cultivars upon PSTVd infection are leaf malformations, chlorotic/necrotic lesions, growth reduction and significant loss in yield. In the present study, successful PSTVd infection on tomato plants was observed by detection of the necrotic lesions in systemic leaves. The trypan blue staining technique assesses the cell viability of leaves. The dead cells are visualized as coloured in blue. In Figure 1, the leaves infected with PSTVd KF440-2 or PSTVd G241-C showed blue-stained areas, indicating that PSTVd induced necrosis. The PSTVd C208-U variant did not show PSTVd-specific necrosis at 28 d.p.i.; such was observed at a later stage (close to 35 d.p.i). The PSTVd C227-U variant did not induce necrosis and was confirmed as non-infectious on tomato plants.

### Northern blot analysis to detect PSTVd(+) RNA in systemic leaves of tomato plants

**Figure 2.**
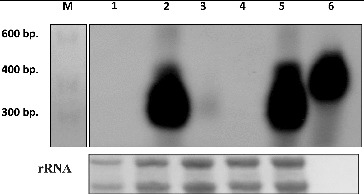
Northern blot analysis to detect PSTVd infection with *P. ramosa* PSTVd variants on systemic leaves of tomato plants. Lane M: RNA Ladder 0.1–1 kb (USB); Lane 1: mock-inoculated; Lane 2: infected with PSTVd variant G241-C; Lane 3: infected with PSTVd variant G208U; Lane 4: infected PSTVd variant C227U; Lane 5: infected with PSTVd KF440-2; Lane 6: *in vitro* synthesized PSTVd(+) RNA. rRNA was used as loading control.

Northern blot analysis showed a size-specific PSTVd signal in tomato plants infected with PSTVd KF440-2 and the PSTVd variant G241-C (Figure 2). A positive but less intensive signal was observed in tomato plants infected with the PSTVd C208-U variant. No PSTVd specific signal was found in the systemic leaves of tomato plants infected with the PSTVd C227-U variant (Figure 2). However, this variant was reported to be able to replicate in the inoculated leaves [23] and was further studied.

### Detection of *P. ramosa* PSTVd variants in floral organs of tomato plants

#### RT-PCR analysis

**Figure 3.**
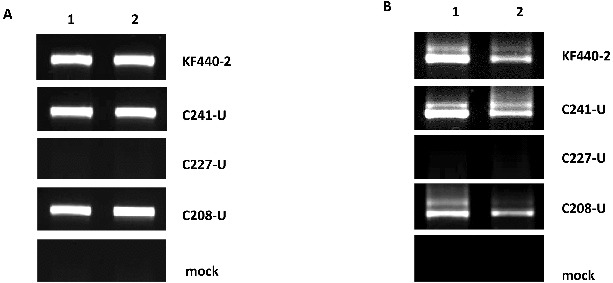
RT-PCR to detect PSTVd(+) strand (a) and PSTVd(−) strand (b) in floral organs of tomato plants infected with PSTVd KF440-2 isolate and *P. ramosa* variants and mock-inoculated tomato plants. Lane 1: sepals; Lane 2: petals. The PSTVd variants used for infection are indicated on the right of each panel.

The results from the PSTVd-specific RT-PCR analysis are shown in Figure 3. PSTVd(+)-specific products were detected both in the sepals and petals of tomato plants infected with the KF440-2 isolate and *P. ramosa* variants G241-C and C208-U (Figure 3(A)). PSTVd(−)-specific signals as well as replication intermediates of lower electrophoretic mobility were detected in the sepals and petals of tomato plants infected with the KF440-2 isolate and *P. ramosa* variants G241-C and C208-U (Figure 3(B)). No PSTVd signals were observed either in the sepals and petals of tomato plants infected with *P. ramosa* C227-U or in the mock-inoculated tomato plants (Figure 3(A) and 3(B). Our experimental system showed for the first time that the PSTVd KF440-2 isolate and two of the *P. ramosa* PSTVd variants G241-C and C208-U have the ability to replicate in all floral organs of mechanically infected tomato plants, which had been reported only in transgenic *N. benthamiana* that expressed cDNA of PSTVd intermediate strain and was not observed in the same mechanically inoculated system [7]. Moreover, our results revealed both replication ability and trafficking competence of the KF440-2 isolate and *P. ramosa* variants G241-C and C208-U in the floral organs of tomato plants.

#### Northern blot analysis

**Figure 4.**
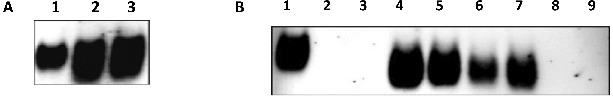
Northern blot analysis to detect PSTVd in floral organs of tomato plants. (a) Detection of PSTVd KF440-2. Lane 1: *in vitro* synthesized PSTVd(+) transcript; Lane 2: PSTVd KF440-2 in sepals; Lane 3 PSTVd KF440-2 in petals. (b) Detection of *P. ramosa* PSTVd variants. Lane 1: *in vitro* synthesized PSTVd(+) transcript; Lane 2: mock-inoculated sepals; Lane 3: mock-inoculated petals; lane 4: G241-C variant in sepals; Lane 5: G241-C variant in petals; Lane 6: C208-U variant in sepals; Lane 7: C208-U variant in petals; Lane 8: C227-U variant not detected in sepals; Lane 9: C227-U variant not detected in petals.

To confirm the RT-PCR analysis, we applied the Northern blot hybridization technique. PSTVd specific signals were detected both in the sepals and the petals of tomato plants infected with the KF440-2 isolate and the G241-C and C208-U PSTVd variants (Figure 4). There was no PSTVd signal in the mock-inoculated tomato plants and the PSTVd C227-U variant. Thus, the results from the Northern blot analysis confirmed the RT-PCR data.

### Sequence analysis of PSTVd progeny

Analysis of the PSTVd populations in the sepals and petals of tomato plants inoculated with the KF440-2 isolate and the studied *P. ramosa* variants was performed by bidirectional sequencing of two independently obtained complete cDNA clones from two separate floral organs. We revealed stable maintenance of the KF440-2 isolate in the sepals and petals of tomato plants. The PSTVd G241-C and C208-U sequence variants were also retained in the sepals and petals of tomato plants. However, parallel to parental genomes, we observed a reversion to the wild-type KF440-2 sequence in petals infected with the PSTVd G241-Cvariant and in sepals and petals infected with the PSTVd C208-U variant. These sequencing results are in support of a heterogeneous nature of the PSTVd population in tomato plants infected with *P. ramosa* variants. The specific nucleotide change characteristic of these variants could be implicated in their replication ability and competence for trafficking towards the floral organs of the infected tomato plant. The PSTVd variants obtained from *P. ramosa* were previously reported to adopt structures that differ from the wild-type KF440-2 conformation, by using *in silico* RNA folding package (Mfold).[28] The PSTVd C208-U variant has shown the most distorted structure compared to the wild-type PSTVd KF440-2. Our results indicated that the C208-U substitution most likely affects either the movement or the replication capacity of the mutant variant in systemic leaves (Figure 2) but does not compromise its replication in floral organs (Figures 3 and 4).

According to the Mfold prediction tool results, the G241-C variant showed preservation of the KF440-2 secondary structure. Similarly to KF440-2, this variant showed systemic movement and successful replication in sepals and petals of tomato plants (Figures 3 and 4). The PSTVd variant C227-U did not show PSTVd-specific signals in systemic leaves as well as in sepals and petals of tomato plants, which suggests that it does not move from the inoculated leaf towards the flower organs. The C227-U mutant has been reported to be defective in trafficking in systemic leaves of *N. benthamiana* by Zhong et al. [29], who showed that this substitution transforms the A135-C227 loop to an elongated stem and this possibly causes disruption in the interaction with cellular factors that assist different aspects of replication and trafficking.

## Conclusions

In the present study, PSTVd G241-C and C208-U variants isolated from *P. ramosa* were identified in the floral organs of mechanically inoculated tomato plants. We showed that these PSTVd variants successfully replicate both in sepals and in petals. The sequence analysis of the population of their progeny in the floral organs of tomato plants showed retention of these mutations in parallel with sequences that are revertant to the wild-type KF440-2 sequence. The PSTVd C227-U variant was not found in sepals and petals, which suggests that it does not move systemically. This work enriches the knowledge on the systemic trafficking of PSTVd in tomato plants by presenting variants that are able to traffic towards the floral organs without being restricted when moving across different cellular boundaries in the tomato plant.
